# Responding to Cholera in Haiti: Implications for the National Plan to Eliminate Cholera by 2022

**DOI:** 10.1093/infdis/jiy491

**Published:** 2018-09-20

**Authors:** Yodeline Guillaume, Ralph Ternier, Kenia Vissieres, Alain Casseus, Maurice J Chery, Louise C Ivers

**Affiliations:** 1 Center for Global Health, Massachusetts General Hospital, Boston, Massachusetts; 2 Partners In Health/Zanmi Lasante, Boston, Massachusetts and Cange, Haiti; 3 Department of Global Health and Social Medicine, Harvard Medical School, Boston, Massachusetts

Haiti is confronting an 8-year-long cholera epidemic, which until 2017 caused at least 27000 cases annually, with over half of them being severe enough to require hospitalization. Since its emergence in October 2010, the disease has cumulatively sickened more than 810000 Haitians, killed nearly 10000, and become endemic [1]. Other estimates indicate a considerably higher burden, uncaptured by official numbers due to the underreporting of infections and deaths managed outside the healthcare system [2, 3]. Still, between 2010 and 2011, the country accounted for 57% of all cholera incidents and 45% of all related fatalities registered globally, making this epidemic one of the largest in recent history [4].

The explosive spread of cholera was linked to several biosocial factors, not least of which was the population’s initial immunological naïveté to toxigenic *Vibrio cholerae*, having no prior documented exposure to the disease before 2010 [5]. Cholera was inadvertently introduced into Haiti’s longest river through the improper disposal of untreated sewage from a United Nations peacekeeping base in Mirebalais in the Central Plateau and would subsequently propagate to all other regional departments [6]. The bacterial strain (serogroup O1, serotype Ogawa, biotype El Tor) causing the epidemic also contained a classical type of cholera toxin associated with more severe diarrhea and protracted outbreaks [7, 8].

Further exacerbating the situation were Haiti’s pre-existing deficiencies in water, sanitation, hygiene (WASH), and healthcare infrastructure. At the start of the epidemic, only an estimated 54% of Haitians had access to healthcare [9], 62% to an improved water source near their homes, 26% to an improved, nonshared sanitation facility, and 24% to a handwashing station with water and soap [10]. In the Artibonite and Central Plateau departments, where the nonprofit organization Partners In Health and its sister organization in Haiti, Zanmi Lasante (PIH/ZL) operate and have assisted with the cholera response, only 60% and 40% of the population had access to an improved water source, respectively. In both regions, which are 2 of the most cholera-affected departments in Haiti, less than 3% owned an improved toilet [11]. Such conditions facilitated epidemic spread and still continue to play a role in ongoing transmission.

## THE PUBLIC HEALTH RESPONSE TO CHOLERA

Working with the Haitian Ministry of Health (MoH) and other partners, PIH/ZL helped deploy a complementary, comprehensive, and sustained response strategy that called for the use of all immediately available and effective tools to minimize the devastating impact of the epidemic. After 7 years of prevention and control interventions, cholera incidence is at its lowest to date with 1374 cases and 12 deaths recorded between January and April 2018 [1]. This positive evolution has raised cautious hope for attaining the MoH’s national goal of eliminating transmission by 2022 [9]. With less than 4 years remaining to this end date, a review of the emergency response can inform the strategies for meeting this target.

### Case Finding Through Community Engagement and Network of Community Health Workers

**Figure 1.**
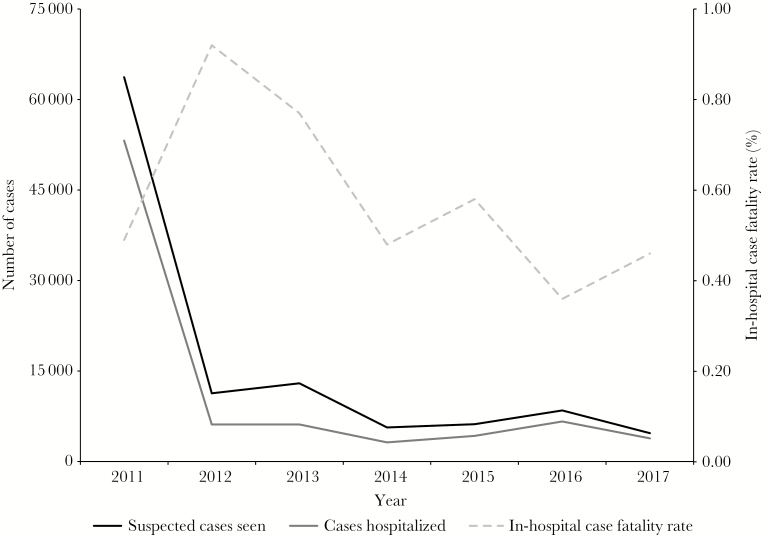
Number of suspected cholera cases seen, number of cases hospitalized, and in-hospital case fatality rate at 12 Partners In Health and Haiti, Zanmi Lasante/Ministry of Health-supported health facilities in the Artibonite and Central Plateau departments, 2011–2017.

Early detection of cases was crucial to decreasing the number of patients who presented too late at health facilities for live-saving treatment and ultimately contributed to maintaining a case- fatality rate (CFR) of less than 1% in the Artibonite and Central Plateau throughout the epidemic (see Figure 1). Building on an existing network of community health workers (CWHs) for delivering health education and care in isolated rural areas, 3300 CWHs were recruited and trained to identify the signs and symptoms of cholera, conduct local outreach, initiate treatment with oral rehydration solution (ORS), and refer cases to health facilities. The training curriculum was developed in collaboration with the MoH and other partners and was designed to be covered over 1 to 3 days [12]. To support case detection, community leaders were also educated on the importance of reporting suspected incidents to CHWs and seeking medical care for sick individuals immediately.

### Early Warning Surveillance Systems Established for Improved Response

As part of Haiti’s national surveillance systems, PIH/ZL compiled and sent cholera morbidity and mortality data from their health facilities to the MoH. The CHWs contributed to these efforts by submitting weekly reports on the number of community cases and deaths. Those working in remote locations were equipped with cellular phones that were specially programmed with a health application to track and send information such as the age, sex, location, symptoms, and dehydration status of cholera patients in real-time [13]. The application was built by PIH/ZL medical informatics team on the open source data collection platform CommCare and included prompts in the local language.

The CHWs were trained on how to use the mobile devices and application to send collected data to a secure, web-hosted database, which was monitored by surveillance staff. From there, reports could be generated for the community health nurse and regional cholera field coordinator responsible for deploying emergency response teams to affected localities. This system enabled the rapid detection of cases and outbreaks, which accelerated interventions such as the distribution of ORS or establishment of oral rehydration posts, reinforcement of cholera prevention and hygiene messages, as well as the provision of materials for water treatment and handwashing in high-risk households and communities [14]. Furthermore, through its partnership with the Haitian MoH, PIH/ZL helped build the diagnostic capacity of 2 public laboratories to confirm suspected cholera cases and strengthen surveillance.

### Case Management Through Delivery of Adequate Care in Appropriate Health Facilities

To help manage the large influx of patients at the height of the epidemic, 11 cholera treatment centers were constructed and 5 additional health facilities outfitted as cholera-specific sites throughout the Artibonite and Central Plateau. The 300 health workers staffing these sites received continuous training on both standard treatment (eg, ORS, intravenous rehydration) and complementary protocols (eg, the use of antibiotics for moderate and severe cases, as well as zinc and vitamin A supplements for children). Decreases in cases and CFRs suggested an important role of this integrated care package, combined with early detection, in reducing suffering and preventable deaths.

With declining morbidity and mortality, the MoH called for the integration of cholera management into the healthcare system, turned cholera-specific facilities into treatment centers for all diarrheal diseases (Centre de Traitement des Diarrhées Aiguës [CTDAs]), and advocated for the care of cholera cases in all health facilities [9]. However, clinical experience indicates that daily cholera case management mostly occurs in the CTDAs. Plans are underway to establish a model CTDA at the public teaching hospital in Mirebalais, with an integrated laboratory and epidemiological surveillance system, to serve as a center of excellence on the delivery of education and care for diarrheal diseases within a larger hospital system.

### Health Education Linked With the Tools for Behavioral Change

In resource-poor settings, the effectiveness of public health messages is contingent on the ability of households to access and/or afford the resources needed for behavioral change. Without the emergency provision of WASH supplies, the poorest households in the PIH/ZL’s catchment areas would have been unable to act on their acquired health knowledge. Therefore, in addition to conducting public hygiene education campaigns via radio and community trainings, the emergency response included the distribution of supplies such as ORS and hygiene kits as well as the disinfection of patients’ home. In addition, some improvements in local water and sanitation infrastructure were undertaken through the construction or rehabilitation of WASH systems at PIH/ZL-supported clinics, hospitals, and schools.

### Psychosocial Support Integrated Into Emergency Care

To address the psychological toll of the epidemic on individuals who experienced cholera-related deaths in their families or suffered from the stigma associated with the illness, counseling services were added as an integral part of response activities. Social worker assistants were trained to help identify cholera patients who would benefit from a 4-session psychosocial support group facilitated by PIH/ZL psychologists [15]. Three of the sessions focused on addressing negative emotions or reactions triggered by the disease and discussing strategies for dealing with them. The fourth session consisted of home visits to assist with the reintegration of survivors into families and communities that were fearful of potential contamination through contact. In addition, because the protocol for handling the corpses of cholera victims often led to precipitous and unceremonious burials without traditional funeral rites, teams of psychologists and social workers held simple memorial services in the affected communities to help people mourn and share remembrances of their loved ones.

### Oral Cholera Vaccination Added to Emergency Toolkit

In collaboration with the Haitian MoH and other partners, we demonstrated the feasibility and effectiveness of complementary, reactive oral cholera vaccination (OCV) programs in epidemic settings, based on a pilot project that vaccinated 50000 individuals in 2 high-risk communities [16]. The evidence from this project and the work of others contributed to the inclusion of OCV as part of the standard response to cholera outbreaks and to the creation of a global OCV stockpile for emergency use. In Haiti, several targeted vaccination campaigns have since been implemented by the MoH to prevent or curb cholera surges among susceptible populations, including almost 1 million people vaccinated in the southern peninsula after the passage of Hurricane Matthew in 2016 and over 100000 in the Central Plateau in 2017.

Together, these interventions mitigated epidemic spreading, but they were insufficient to completely eliminate cholera transmission. Despite an overall trend of declining incidence, transmission still occurs in alternating phases of peak and lull periods driven by seasonal conditions and superimposed outbreaks in hotspots. In 2014, Haiti saw a significant reduction in cases and an opportunity to end the epidemic, but gaps in funding led to the scaling back of both early warning surveillance capacity and emergency WASH interventions in vulnerable communities, likely contributing to a dramatic recrudescence of cholera at the national level in 2015 and 2016 [17].

## IMPLICATIONS FOR HAITI’S NATIONAL CHOLERA ELIMINATION PLAN

The public health response to cholera was largely in line with the Haitian MoH’s national plan, which in itself already contained many of the strategies recently outlined by the Global Task Force on Cholera Control in a roadmap for ending the disease [18]. The PIH/ZL conducted its work within the public sector, supporting MoH facilities in the Artibonite and Central Plateau departments in concert with multisectoral, local, and global stakeholders who shared technical guidance, practical experiences, and resources. With modest investments in public health infrastructure and the strategic cultivation and effective coordination of intersectoral partnerships, similar work could be done by the MoH and its collaborators in other regions of the country. However, in recent years, the Haitian government and its international partners have struggled to mobilize the resources necessary for maintaining and strengthening surveillance systems and improving access to WASH services [19]. Therefore, renewed commitment from all stakeholders is required for the continued, timely detection and treatment of cases and for ensuring consistent progress towards the goal of eliminating cholera transmission in Haiti by 2022. Without sufficient funding and political will, history risks repeating itself and the gains made thus far may be lost to resurging outbreaks similar to those in 2015 and 2016.

## CONCLUSIONS

Greater investments in the health system and public WASH infrastructure remain crucial for the prevention of cholera and other diarrheal diseases and to protect the health of all Haitians. Although development efforts to build and increase access to these systems are being pursued, comprehensive cholera treatment and prevention activities including large-scale OCV campaigns combined with targeted WASH interventions should be used to help protect at-risk communities living in areas known for ongoing transmission. Linking cholera prevention with the provision of routine primary care for these populations would also facilitate the delivery of health education and hygiene promotion during lull periods and subsequently help sustain the behavioral changes that need periodic reinforcement.

## Notes

***Acknowledgments.*** We dedicate this paper to the thousands of Haitian healthcare workers on the frontline of fighting cholera in their communities.

***Financial support.*** This work was funded by grants from the Bill and Melinda Gates Foundation (OPP1148213; to L. C. I.) and the National Institute of Allergy and Infectious Diseases (R01AI099243; to L. C. I.).

***Supplement sponsorship.*** This work is part of a supplement sponsored by the Bill and Melinda Gates Foundation.

***Potential conflicts of interest.*** All authors: No reported conflicts of interest. All authors have submitted the ICMJE Form for Disclosure of Potential Conflicts of Interest.
